# Out-of-Pocket Costs of Treatment Among Employer-Insured Women With Invasive Breast Cancer

**DOI:** 10.1001/jamanetworkopen.2023.1507

**Published:** 2023-03-03

**Authors:** Audrey Hager, Gabriela Gracia, Danielle Rodin, Rena M. Conti

**Affiliations:** 1 Department of Markets, Public Policy, and Law, Boston University, Boston, Massachusetts; 2 Radiation Medicine Program, Princess Margaret Cancer Centre, Toronto, Ontario, Canada; 3 Department of Radiation Oncology, University of Toronto, Toronto, Ontario, Canada

## Abstract

This cross-sectional study examines out-of-pocket costs for the treatment of invasive breast cancer in employer-insured women younger than 65 years.

## Introduction

Oral anticancer prescription drugs are central to breast cancer treatment,^1^ yet high out-of-pocket (OOP) costs are associated with treatment nonadherence and discontinuation.^2,3^ Little is known about OOP costs of treatment associated with invasive breast cancer (in which cancer cells have spread to other areas of the body) among employer-insured women younger than 65 years. This population may face significant financial burdens related to long-term hormonal-based prevention^4^ and enrollment in high-deductible health plans (HDHPs) and consumer-driven health plans (CDHPs). This study examines OOP costs of treatment of invasive breast cancer in employer-insured women younger than 65 years.

## Methods

This cross-sectional study used the national Marative MarketScan database^3^ to identify women 18 to 64 years of age continuously enrolled in a health plan in 2018 with a diagnosis of invasive breast cancer and a claim for at least 1 anticancer drug approved by the US Food and Drug Administration (FDA). Data on race and ethnicity are not collected in the database. Total OOP costs of treatment per patient were calculated as the sum of copayments, coinsurance, and deductibles for pharmaceutical, inpatient, and outpatient claims. Mean oral drug OOP costs were calculated with the same cost variables and standardized to represent a 30-day supply. Costs were inflated to 2022 dollars using the US Bureau of Labor Statistics’ Consumer Price Index for All Urban Consumers. The data were collected from insurers in a Health Insurance Portability and Accountability Act of 1996–compliant manner and are fully deidentified. This study followed the Consolidated Health Economic Evaluation Reporting Standards (CHEERS) reporting guideline and was designated exempt by the Boston University Institutional Review Board for the use of deidentified data.

Statistical analysis used Stata software, version 16 (StataCorp LLC) to estimate the mean annual total OOP costs for treatment per patient per year and mean 30-day OOP cost per drug using standard 2-part logit models. A γ log-link regression model estimated potential correlates in adjusted 30-day drug OOP costs, including year of FDA approval and insurance type. A 1-sided *P* < .05 was considered statistically significant.

## Results

A total of 25 224 women (mean [IQR] age, 53 [48-59] years) with invasive breast cancer diagnosis and claims for 1 or more of 14 oral anticancer drugs were included. A total of 23.1% were HDHP or CDHP insured, and 51.0% had no OOP costs for drugs. Sixteen women had no OOP costs for all care modalities. The total mean estimated annual OOP cost was $1502.23 per patient, with $112.41 (95% CI, $112.40-$112.42) in inpatient costs, $1186.27 (95% CI, $1185.67-$1188.16) in outpatient costs, and $203.55 (95% CI, $203.34-$203.78) in pharmaceutical costs. The OOP costs for outpatient claims were 79.0% of total costs (Figure). Of drug claims, 87.0% were for nonproprietary drugs, and the mean OOP cost of filling a 30-day supply of individual drugs ranged from $0.58 (95% CI, $0.57-$0.60) for tamoxifen to $137.58 (95% CI, $134.08-$141.07) for palbociclib (Table). Regression modeling showed a positive, statistically significant correlation between drug costs and more recent year of FDA approval. Patients with CDHPs and HDHPs had higher OOP costs compared with those insured with preferred provider organization and exclusive provider organization plans (β = −0.031, SE = 0.06; *P* = .001).

**Figure.**
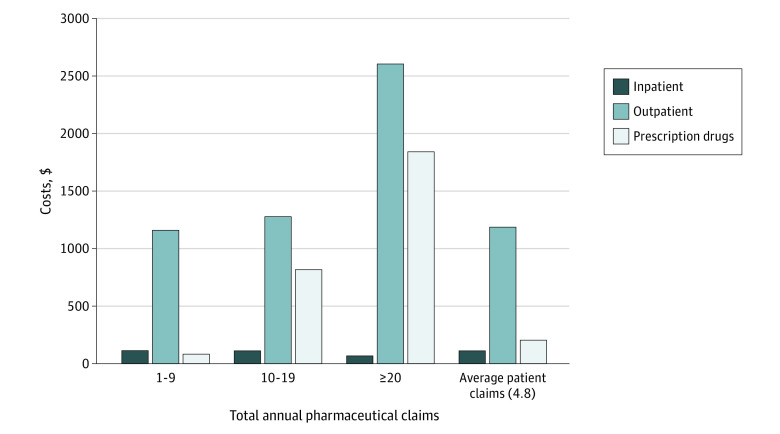
Annual Out-of-Pocket Costs by Type of Care and Intensity of Pharmaceutical Claims for Women Prescribed Oral Breast Cancer Medications in 2018 Data were extracted from the 2018 Marative MarketScan Database. Inpatient and outpatient claims are characterized by all claims that included *International Statistical Classification of Diseases, Tenth Revision, Clinical Modification* codes for invasive breast cancer. A total of 51.0% of the sample had no out-of-pocket costs for prescription drugs, and 16 women in the sample did not have any out-of-pocket costs for all modalities of care. Prescription drug claims characterized by all claims with a National Drug Code for a US Food and Drug Administration–approved drug for breast cancer if the patient also has an inpatient or outpatient diagnosis of cancer. All numbers are inflated to 2022 Bureau of Labor Statistics’ Consumer Price Index for Urban Consumers.

**Table.** Claims per Prescription Drug per Patient and Mean Annual OOP Costs by Prescription Drug for 121 787 Women Undergoing Invasive Breast Cancer Treatment with Oral Anticancer Drugs in 2018^a^

Drug	Total No. of claims	No. of claims per drug per patient, mean (SD)^b^	Annual OOP costs per person, median (95% CI), $^c^
**Available only as proprietary (patented)**			
Abemaciclib	1228	7.8 (4.1)	51.52 (50.04-52.99)
Letrozole and ribociclib	170	7.6 (3.9)	21.01 (19.10-22.95)
Lapatinib	735	9.3 (8.1)	54.94 (52.85-52.77)
Olaparib	503	6.4 (3.9)	51.67 (50.58-52.77)
Palbociclib	11 918	10.0 (4.9)	137.58 (134.08-141.07)
Ribociclib	489	8.9 (6.0)	37.89 (37.89-37.89)
Talazoparib	3	1.0 (NA)	NA^d^
Everolimus	1281	10.4 (6.2)	74.25 (73.76-74.75)
Total proprietary	16 327	7.6 (5.3)	NA
**Available as nonproprietary**			
Capecitabine^e^	8852	12.1 (8.8)	19.66 (19.63-19.70)
Exemestane^e^	13 062	4.6 (3.4)	12.22 (12.22-12.25)
Letrozole^e^	14 974	3.8 (3.5)	3.30 (3.30-3.31)
Anastrozole	22 150	4.1 (2.8)	3.83 (3.79-3.84)
Megestrol acetate	122	4.5 (4.4)	3.48 (3.33-3.63)
Tamoxifen citrate	46 300	4.4 (3.4)	0.58 (0.57-0.60)
Total nonproprietary	105 460	5.6 (4.4)	NA

Abbreviations: NA, not applicable; OOP, out-of-pocket.

^a^ Data from this table were extracted from the 2018 Marative MarketScan Database. All mean OOP cost estimates are adjusted for inflation in 2022 by the US Bureau of Labor Statistics. Claims were determined by individual National Drug Codes for oral anticancer prescription drugs approved by the US Food and Drug Administration.

^b^ Mean frequency of how often these oral anticancer prescription drugs were prescribed in 2018 per patient.

^c^ These estimates have a 95% CI based on their estimates in the γ log-link regression model.

^d^ These oral anticancer prescription drugs had claims for both their nonproprietary and proprietary versions in the Marative MarketScan database.

^e^ This oral anticancer prescription drug did not have enough claims to make statistically significant cost estimates.

## Discussion

Among a prevalent sample of younger women with invasive breast cancer and employer-sponsored insurance who filled prescriptions for oral anticancer drugs in 2018, most used nonproprietary drugs and had limited OOP costs. Outpatient-related OOP costs were larger than drug costs, and OOP costs were higher among those using branded or more recently launched drugs.^5^ Women insured by HDHPs and CDHPs had higher OOP costs compared with those with more generous coverage.

This study has some limitations. The OOP costs are an underestimate because many patients will also be treated for other comorbidities^6^ and complications related to treatment. We were also unable to estimate costs by cancer treatment phase^6^ or clinical history and among those 65 years or older. Recently passed federal policies seeking to reduce prescription drug prices among Medicare-insured patients could be complemented by voluntary employer or insurer efforts and state legislation to reduce OOP costs for commercially insured patients with cancer across care modalities.

## Notes

Supplement.
Data Sharing Statement
