# Supplementary material for: Out-of-Pocket Costs of Treatment Among Employer-Insured Women With Invasive Breast Cancer
Source: JAMA Netw Open. 2023 Mar 3;6(3):e231507. doi: 10.1001/jamanetworkopen.2023.1507 (PMC9984972; doi:10.1001/jamanetworkopen.2023.1507)
Supplement: Supplement. — Data Sharing Statement [file jamanetwopen-e231507-s001.pdf]

Data Sharing Statement

Hager. Out-of-Pocket Costs of Treatment Among Employer-Insured Women With Invasive Breast Cancer. *JAMA Netw Open*. Published March 03, 2023.
doi:10.1001/jamanetworkopen.2023.1507

Data
Data available: No

Additional Information
Explanation for why data not available: The data are available under license from a private data vendor. We would be happy to make public our code and variable definitions if requested by the editors, reviewers or readers.
